# Transformation of Australian Community Pharmacies Into Good Clinical Practice Compliant Trial Pharmacies for HIV Pre-Exposure Prophylaxis

**DOI:** 10.3389/fphar.2019.01269

**Published:** 2019-11-07

**Authors:** Luxi Lal, Kathleen Ryan, Iris Yi Liu, Brian Price, Timmy Lockwood, Ivette Aguirre, Peter Slobodian, Ada Lam, Manoj Vassan, Kie Lim, John Silverii, Joseph Tesoriero, Johnny Phu, Wan Lim, Bharathy Naidoo, Nick Russell, Matthew Rundle, Rowan Sewell, Craig Cooper, Alexander Hardman, Martin Quinn, Anne Mak, Edwina J. Wright

**Affiliations:** ^1^Alfred Health, Melbourne, VIC, Australia; ^2^Burnet Institute, HIV Elimination Program, Melbourne, VIC, Australia; ^3^Department of Infectious Diseases, Alfred Health, Monash University, Melbourne, VIC, Australia; ^4^Clinical Trials Pharmacy, Royal Adelaide Hospital, Adelaide, SA, Australia; ^5^Prahran Central Pharmacy, South Yarra, VIC, Australia; ^6^John Silverii’s Pharmacy, Fitzroy North, VIC, Australia; ^7^HealthSmart Pharmacy Alfred, Melbourne, VIC, Australia; ^8^Newton & Leung Pharmacy, Collingwood, VIC, Australia; ^9^Bain & Co. Pharmacy, St Kilda, VIC, Australia; ^10^Centre Pharmacy, Central Market Arcade, Adelaide, SA, Australia; ^11^Epic Pharmacy New Town, Hobart, TAS, Australia; ^12^Epic Pharmacy Kings Meadows, Launceston, TAS, Australia; ^13^The Peter Doherty Institute of Infection and Immunity, University of Melbourne, Melbourne, VIC, Australia

**Keywords:** pre-exposure prophylaxis, pre-exposure prophylaxis expanded, pharmacy, clinical trials, human immunodeficiency virus prevention, prophylaxis

## Abstract

**Background:** In Australia, clinical trial drugs are conventionally dispensed through clinical trial pharmacies only, while community pharmacies dispense drugs approved by Australia’s regulatory body. A large HIV pre-exposure prophylaxis study aimed to deliver clinical trial drug through community pharmacies to improve convenience and mimic real world prescribing. This paper describes the process of making community trials compliant with good clinical practice and reports outcomes of delivering clinical trial drug through community pharmacies.

**Methods:** Eight community and four clinical trial pharmacies across three Australian states were approached to participate. A good clinical practice checklist was generated and pharmacies underwent a number of changes to meet clinical trial pharmacy requirements prior to study opening. Changes were made to community pharmacies to make them compliant with good clinical trial practice including; staff training, structural changes, and implementing monitoring of study drug and prescribing practices. Study drug was ordered through standard clinical trial processes and dispensed from study pharmacies by accredited pharmacists. Throughout the trial, record logs for training, prescriber signature and delegation, temperature, participant, and drug accountability were maintained at each pharmacy. The study team monitored each log and delivered on-site training to correct protocol variations.

**Results:** Each pharmacy that was approached agreed to participate. All community pharmacies achieved good clinical practice compliance prior to dispensing study drug. Over the course of the study, 20,152 dispensations of study drug occurred, 83% of these occurred at community pharmacies. Only 2.0% of dispensations had an error, and errors were predominantly minor. On five occasions a pharmacist who was not accredited dispensed study drug.

**Conclusions:** Community based pharmacies can undergo training and modifications to achieve good clinical practice compliance and dispense clinical trial study drug. Community based pharmacies recorded few variations from study protocol. Community based pharmacies offer a useful alternative to clinical trial pharmacies to increase convenience for study participants and expanded use of these pharmacies should be considered for large clinical trials, including HIV prevention trials.

## Introduction

Clinical trials are essential to the evaluation and approval of novel therapeutic agents, however a number of barriers limit clinical trial participation (Bower et al., 2014). Population groups including people who are culturally and linguistically diverse, people living in regional/remote areas, youth, and indigenous populations are underrepresented in clinical trials (Schmotzer, 2012; Bower et al., 2014; Curran et al., 2015; Ernst and Young, 2016). In Australia, many trials struggle to meet recruitment and retention targets (Ernst and Young, 2016).

In Australia, clinical trials are conventionally conducted through tertiary hospitals with study drug dispensed by the hospitals’ clinical trial pharmacies. Hospital-based clinical trial pharmacies dispense investigational drugs that have not been approved by the Therapeutic Goods Administration (TGA) and are required to adhere to state-based legislation and the relevant research governance framework (Australian Government Department of Health TGA, 2018). Clinical trial drugs are typically dispensed at no cost to the study participant. Conversely, community pharmacies (also known as retail pharmacies) are only permitted to dispense drugs that have been approved by the TGA, must adhere to federal legislation in relation to pharmacy dispensing, and individuals are required to pay for each drug dispensing event [for drugs listed on Australia’s Pharmaceutical Benefits Scheme (PBS) in 2018: $39.50 Australian dollars (AUD) or AUD$6.40 for concession (Australian Government Department of Health, 2018)].

HIV pre-exposure prophylaxis (PrEP) is the use of co-formulated tenofovir and emtricitabine by HIV-negative people to prevent HIV acquisition (Grant et al., 2010). Randomized clinical trials and demonstration projects have demonstrated that PrEP is highly efficacious and effective at preventing the sexual and injection transmission of HIV when high medication adherence is achieved (Grant et al., 2010; Baeten et al., 2012; Thigpen et al., 2012; Choopanya et al., 2013; Grant et al., 2014; Molina et al., 2015; McCormack et al., 2016). HIV PrEP was registered on Australia’s TGA in 2016 (Australian Government Department of Health TGA, and biologicals, 2016), however it was not listed on the PBS until April 2018 (Health AGD of. Pharmaceutical Benefits Scheme (PBS) | For PBS Prescribers [Internet], 2018). Prior to the PBS listing of PrEP, access to PrEP was primarily through participation in local clinical trials that aimed to demonstrate the clinical effectiveness (Lal et al., 2017; Rodriguez et al., 2018;Zablotska et al., 2018) and the population level prevention benefit of PrEP (AVAC, 2015; Grulich et al., 2018;Rodriguez et al., 2018; Ryan et al., 2018; Western Australia AIDS Council, 2018).

The pre-exposure prophylaxis expanded (PrEPX) study was a multi-site, one-armed, open-label, population-level PrEP intervention study undertaken in Australia. The primary objective of the PrEPX study was to measure the change in HIV incidence at a population-level (Ryan et al., 2018). The PrEPX study originated in Victoria providing 3,800 study places, and expanded to include South Australia (650 study places) and Tasmania (100 study places). PrEPX was designed to mimic the anticipated real world conditions of PrEP prescribing that would be in place if PrEP were to receive PBS approval. Real world conditions included: attending primary care and sexual health services for study visits, having study drug dispensed at community pharmacies which included paying conventional clinical service and PBS co-payments for drug dispensing.

**Figure 1:**
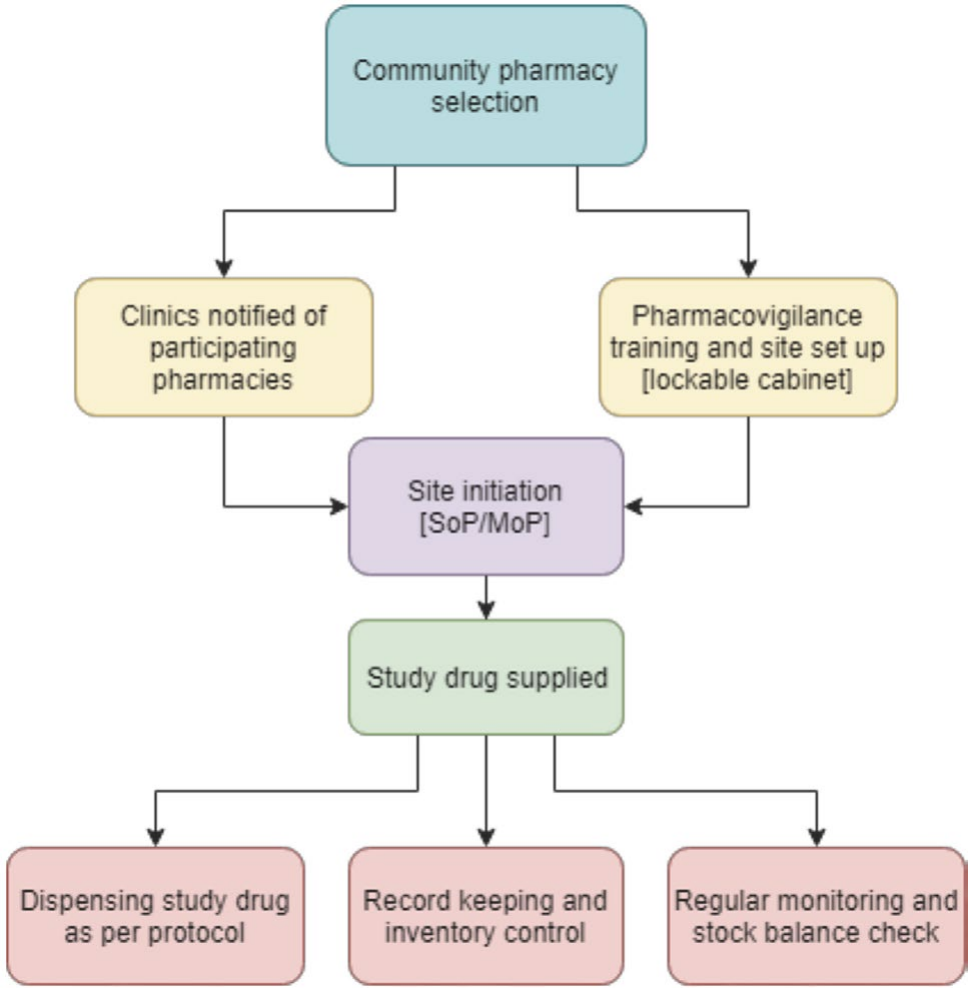
Flow chart of the set up process for involvement of community pharmacies (SoP—Standard Operating Procedure; MoP—Manual of Operations).

The PrEPX study was the first clinical trial undertaken in Australia whose protocol design permitted community pharmacists to dispense clinical trial medications in accordance with International Conference on Harmonization-Good Clinical Practice (ICH-GCP) guidelines (ICH-GCP ICOHGCP, 1996; Abraham, 2009; Australian Government Department of Health TGA, 2018). A smaller Victorian PrEP demonstration study (Lal et al., 2017) and PrEP demonstration studies in other Australian jurisdictions did not utilize community pharmacies for prescribing, nor require PBS co-payments (Grulich et al., 2018; Rodriguez et al., 2018; Western Australia AIDS Council, 2018; Zablotska et al., 2018). In this paper we describe how clinical trial dispensing was implemented through community pharmacies across three Australia states, Victoria, South Australia, and Tasmania. We describe the consultation with community pharmacists, steps required to meet clinical trial pharmacy requirements, ongoing monitoring, and outcomes of utilizing community pharmacies to dispense clinical trial drug (See **Figure 1**).

## Methods

### Study Overview

The PrEPX protocol has been described elsewhere (Ryan et al., 2018). Here, we detail the implementation and outcomes of the study’s community pharmacy dispensing of study drug. The PrEPX study was a population level demonstration study aimed to recruit 2,600 individuals at risk of HIV, in order to show a decline in HIV incidence (Traeger et al., 2019).

The study design required participants to attend study clinics, including hospital, primary care, and/or sexual health services, for quarterly HIV and sexually transmitted infection testing and provision of a study drug prescriptions. Participants then attended participating study pharmacies to have study-specific prescriptions dispensed. Participants could attend any of the participating PrEPX pharmacies to have study drug dispensed. Community pharmacies participating in PrEPX underwent specific modifications and training to meet clinical trial pharmacy requirements to participate as a PrEPX clinical trial pharmacy.

The PrEPX pharmacy team included three key team members—lead pharmacist, pharmacy manager, and pharmacy monitor. The PrEPX lead pharmacist was registered with the Pharmacy Board of Australia, and appointed from the Alfred Clinical Trials Pharmacy. The PrEPX lead pharmacist held a bachelor’s degree in pharmacy with more than 30 years experience of working in clinical trials. The PrEPX pharmacy manager had completed a bachelor of pharmacy degree and the pharmacy monitor held a life sciences degree. Alfred Health Clinical Trials Pharmacy provided pharmacy oversight, in line with ICH-GCP guidelines and relevant federal and state-based legislation. Registered, board certified community pharmacy owner(s) were trained and delegated PrEPX dispensing duties as per the approved study protocol.

Funding for PrEPX was announced in January 2016. The PrEPX study opened for enrolment on 26 July 2016 in Victoria, and was funded to enroll 2,600 participants; subsequently, further funding permitted the study to enroll 3,800 participants however the study went on to enroll 4,275 participants in Victoria. The PrEPX study closed to enrolment in Victoria when PrEP was subsidized on the PBS on April 1^st^ 2018 in Victoria. The study expanded to South Australia on 15 May 2017 where 650 participants were enrolled and to Tasmania where 100 participants were enrolled until the study closed to enrolment on 30 June 2018 in South Australia and Tasmania, in accordance with the approved protocol.

### Pre-Study Set Up

A pharmacy compliance group was established at Alfred Health to oversee the pharmacy component of the PrEPX study. The PrEPX pharmacy team was responsible for engaging with, training, and supervising PrEPX dispensing over the course of the study, in all participating states.

The PrEPX pharmacy team, created a checklist for GCP compliance and regulatory dispensing requirements for a community pharmacy acting as a clinical trial pharmacy. Key features of a clinical trial pharmacy are 1) drug storage, 2) record keeping, 3) monitoring of stock, 4) destruction of drugs if permitted by the protocol, 5) assistance with adherence to study protocol, 6) counseling of participants and monitoring medication adherence, 7) and provision of information to participants (Abraham, 2009; Australian Government Department of Health TGA, 2018).

Community pharmacies in close proximity to PrEPX study clinics were approached and invited to participate as a clinical trial pharmacy in the study. A brief survey was sent out to selected pharmacies to determine their current practices, and to identify gaps in clinical trial pharmacy requirements. The PrEPX pharmacy team worked with individual pharmacies to address each identified gap so that pharmacies could dispense clinical trial study drug, in accordance with ICH-GCP requirements (ICH-GCP ICOHGCP, 1996; Abraham, 2009), as per **Supplementary Figure 1**.

#### Structural Factors Implemented

Ambient temperature conditions are not routinely monitored or recorded within community pharmacies in Australia. Pharmacies that did not have temperature monitoring were provided with a testo automated temperature monitoring system (Testo Pty Ltd., 2015) at no cost to the pharmacy. This device was selected as it had been used successfully within Alfred Health Hospital Pharmacies. An active Wi-Fi connection was required to activate individual temperature monitors at each pharmacy. Temperatures were continuously recorded during the study, sending automatic hourly notifications to the secure PrEPX Cloud account. The minimum and maximum temperatures (15°C, 30°C) were set on each device, in line with the study drug storage conditions recommend by the drug manufacturer (Mylan Health Pty Ltd., 2014). In the event of temperatures exceeding the minimum and maximum, an alarm was automatically sent *via* text message to the PrEPX pharmacy manager and the site pharmacist. Hospital-based clinical trial pharmacies are conventionally connected to back up generators, however back-up generators were not purchased for this study as it was determined that pharmacy ambient temperature could be corrected in a timely manner.

The PrEPX study supplied individual, lockable cabinets for study drug storage to participating community pharmacies as needed. The lockable cabinets were placed in the dispensary to comply with GCP requirements that study drug be stored in a restricted access area, under lock and key (Abraham, 2009; Australian Government Department of Health TGA, 2018).

#### Training

GCP training was provided to all study pharmacists who did not have current GCP certification (Whitehall Training, 2016). The TransCelerate online training course (Whitehall Training, 2016) was used and all study pharmacists were required to achieve at least 85% correct responses and provide a certificate of competency to PrEPX pharmacy manager prior to dispensing PrEPX study medications. Pharmacists were reimbursed $350AUD and provided six (Australian Government Department of Health, 2018) Continuing Professional Development points for successfully completing the GCP course (Pharmacy Board of Australia, 2018). Pharmacists who successfully completed GCP training were required to attend a site initiation visit, at which the study protocol and implementation were described. Site pharmacists who were not GCP certified were not permitted to dispense PrEPX study drug, in accordance with Australian Clinical Trial Regulations (Australian Government Department of Health TGA, 2018).

#### Study Prescription

A PrEPX study prescription form was designed by The PrEPX pharmacy team. The prescription form collects all data required in a clinical trial prescription and is consistent with standard practice at the Australian Clinical Trial pharmacies. The prescription form was printed on bright yellow paper to distinguish it from regular PBS prescriptions (Health AGD of. Pharmaceutical Benefits Scheme (PBS) | For PBS Prescribers [Internet], 2018; Australian Government Department of Health.). The PrEPX prescription form included participant name, date of birth, address, participant study number, study visit number or visit month, allergies, consent yes/no, Medicare number, concession number (if applicable), name of prescriber, prescribing date, treatment date, and signature of prescriber (**Supplementary Figure 1**). All PrEPX prescriptions were required to be filled out by hand, by the PrEPX prescriber, using only black ink.

### Study Protocol

#### Ordering Study Drug

The PrEPX study purchased generic co-formulated tenofovir disoproxil fumarate and emtricitabine from Mylan Pharmaceuticals. PrEPX study drug was delivered from Mylan to a centralized, TGA approved distribution and warehousing service for controlled substances, pharmaceutical packaging professionals (PPP), then onto pharmacies as required. PrEPX study drug was supplied to the study pharmacies at no cost to the pharmacy.

Site pharmacists emailed orders for PrEPX study drug to the pharmacy manager in batches of orders consisting of 300 bottles, (3 month supply for 100 prescriptions). The pharmacy manager reviewed all PrEPX study drug orders and emailed approved orders to PPP. Orders were dispatched directly from PPP to participating pharmacies and upon receipt of the PrEPX pharmacy manager approval. Site pharmacists were required to complete a receipt form, confirming the number of bottles received, batch number, and expiration date. The pharmacy manager maintained a log of all orders to generate estimates of dispensing and stock.

#### Dispensing

PrEPX participants were prescribed 3 months (90 days) of study drug at each study visit. Pharmacists were permitted to charge participants the current PBS co-payment fee at each dispensing event (Australian Government Department of Health, 2018). Dispensing fees served as remuneration for the pharmacies’ work in dispensing the study drug, including the required clinical trial record keeping. Dispensing fees could be waived at the pharmacists’ discretion.

The baseline study prescriptions had to be dispensed and collected within 7 days of the treatment date written on the prescription. This design was requisite to prevent a participant from filling the prescription beyond 7 days after the treatment date in case they acquired HIV between the clinic visit and collecting their script; in which case they could have remained undiagnosed with HIV and commenced two-drug treatment for HIV, which is inadequate. For the same reason the participants’ follow-up prescriptions had to be dispensed and collected within 21 days (conventional prescriptions in Australia are valid for 12 months). In addition the limited time between prescribing and filling PrEPX prescriptions was designed to ensure participants had adequate supply of study drug for daily dosing, as per the approved study protocol. In the event of a participant attending a pharmacy outside of these date ranges, the site pharmacist was instructed to contact the PrEPX pharmacy manager to request permission to dispense on a case-by-case basis.

### Monitoring During the Study

#### Monitoring Prescribing

A check box was printed at the bottom of each PrEPX study prescription, to be completed by the dispensing pharmacist to ensure each step of the dispensing process was executed (**Supplementary Figure 1**). The check boxes were 1) dispensing record checked, 2) prescribers’ name and signature listed in delegation log, 3) dispensed medications checked by, 4) handed out by, 5) received by patient. The check boxes required initialing and dating by the authorized delegated pharmacist for compliance (**Supplementary Figure 1**).

The PrEPX pharmacy monitor reviewed PrEPX prescriptions on site at all participating PrEPX pharmacies. The pharmacy monitor completed prescription data entry and data maintenance in accordance with the approved study protocol. Every dispensed PrEPX prescription was checked by either the pharmacy monitor, or pharmacy manager for completion and accuracy in accordance with the key performance indicator checklist. Refer to the sub-section pharmacy monitoring for full details (**Supplementary Figure 2**). Any discrepancies were discussed with the pharmacist and rectified accordingly.

#### Record Logs

Record logs were provided to PrEPX study pharmacies to record practices in line with clinical trial pharmacy practice (**Table 1**). Each log was printed in color to improve ease of use. The PrEPX pharmacy manager was on call to answer issues related to dispensing and completion of record logs over the course of the study. The signed training, signature, and delegation logs were authorized by the principal investigator for GCP compliance.

**Table 1:** Characteristics of pre-exposure prophylaxis expanded study pharmacies.

		Total	Community/retail pharmacy	Hospital-based clinical trials pharmacy
**Total number**		12	8	4
**Number per state**	Victoria	8	5	3
	South Australia	2	1	1
	Tasmania	2	2	0
**Open after 5 pm on weekdays**	Yes	8	8	0
	No	4	0	4
**Open on weekend**	Yes	8	8	0
	No	4	0	4
**Distance from closest PrEPX study clinic**	Meters (range)	0.05–2.0 kms	0.05–2.0 kms	0.05 km
**Number of PrEPX prescriptions filled**	N	20,152	16,724	3,428
**Number of study pharmacists onsite**	N (range)	2–6	2	3
**Charged PrEPX participants PBS co-payment**		12	8	4

PrEPX-pharmacy training logs, signature, and delegation logs were produced in line with the Australian clinical trial handbook (Australian Government Department of Health TGA, 2018). The training log included data of site initiation visit and was signed and dated by pharmacists who attended the initiation. The signature and delegation logs included the name and signature of each pharmacist at each pharmacy that was authorized to dispense PrEPX study drug. Copies of the training and delegation logs were stored electronically by the PrEPX pharmacy team, and in hard copy format at each PrEPX participating pharmacy, as per GCP requirements (**Table 1**).

The prescriber signature and delegation logs were completed by all PrEPX prescribers and authorized by the study’s principal investigator. Prescriber signature logs included names and signatures of approved prescribers and the duration for which they were permitted to prescribe study drug for the purpose of the trial. Prescriber signature logs ensured study prescriptions were written and signed only by authorized PrEPX prescribers. Prescriber logs were provided to all pharmacies in hard copy format and electronically, *via* email. Prescriber signature and delegation logs were updated and supplied to study pharmacies when new PrEPX prescribers joined the study (**Supplementary Figure 3**).

Template temperature logs were supplied to study pharmacies to manually record daily temperatures as a back-up, in the event of temperature monitoring failure with the testo system. Temperature logs included date of entry and both the minimum and maximum temperatures displayed within a 24-h period. There were no temperature monitoring failures at any of the PrEPX pharmacy sites over the course of the study. The PrEPX pharmacy team provided PrEPX pharmacies written advice on how to manage any potential temperature excursion. All temperature excursions were to be reported to the PrEPX pharmacy manager for investigation. All PrEPX stock that was subject to investigation due to a temperature excursion was required to be placed in quarantine and not dispensed to participants until disposition was determined by the PrEPX study management team.

Participant logs were completed by PrEPX pharmacists at a participant’s first prescription dispensation at that pharmacy. Participant logs included dispensing date, participant name, and date of birth (DOB) or medical record number (MRN), participant study number, and consent (**Supplementary Figure 4**).

Drug accountability logs were completed by PrEPX pharmacists at each drug dispensing event. The drug accountability log recorded receipt of study drug, dispensing details, study drug returns, and balance of study drug product (**Supplementary Figure 5**). Date, visit number or month of study visit, participant ID, quantity (bottles), and initials of an authorized GCP trained pharmacist, confirming dispensing details were required to be completed for every PrEPX prescription dispensed at each participating pharmacy. The number of bottles of study drug remaining was recorded in the balance column after the dispensing details. The returns column, recorded the date, quantity of bottles, or number of tablets returned, initials of study pharmacist.

#### Study Team Monitoring

Regular monitoring by the pharmacy monitor or pharmacy manager, using key performance indicators for procedural deviations, drug storage, temperature excursions, and drug accountability accuracy were conducted monthly for the first 6 months of the study, then every 2–3 months thereafter. However, pharmacies that dispensed greater than 300 study prescriptions per month continued to be monitored monthly, or fortnightly for those pharmacies dispensing more than 500 prescriptions per month.

Pharmacy monitors undertook monitoring using a checklist, which was devised by the PrEPX pharmacy team. The monitoring checklist included indicators about a prescribing event: consent checked, study ID recorded, prescription signed by an authorized PrEPX prescriber, date study drug collected (for baseline prescriptions within 7 days, and for follow-up prescriptions within 21 days). The monitor also recorded completion of dispensing check boxes; all entries initialed and dated correctly in the drug accountability log, stock count balance, batch numbers, and page numbers filled out correctly on each page of the accountability logs, single line entries on each log, logs in date order, prescriptions filed in chronological order for review. Structural elements that were monitored were study drug stored in the designated, locked cabinet/drawer, and temperature records, which were checked for excursions using the PrEPX pharmacy monitoring checklist (**Supplementary Figure 2**).

Every dispensed PrEPX prescription was checked for completion and accuracy in line with clinical trial pharmacy requirements and the key performance indicator checklist (**Supplementary Figure 2**) Following study monitoring, the participating pharmacy was emailed items to action and/or issues to be resolved. A report from each monitoring visit was presented to the monitored pharmacy and the PrEPX pharmacy team.

### Data Collection and Reporting

Data in this paper include description of participating pharmacies, outcomes of training, and structural improvements required to meet GCP guidelines. Dispensing log data (date range) reports the number of prescriptions dispensed by state and pharmacy type (community/hospital). Monitoring data from the drug accountability logs and participant logs (July 2016–June 2018) reported the number of errors that required follow-up and described the types of issued rectified.

### Ethics

This project was approved by the Alfred Hospital Health Research Ethics Committee for the study to be conducted in Victoria and South Australia (AH/HREC16/100). Separate ethics approval was sought and granted from the University of Tasmania for the PrEPX study to be conducted in Tasmania (H0016607). The study was registered on the Australian and New Zealand Clinical Trials Registry (ACTRN12616001215415). All study participants provided signed consent at enrollment.

## Results

### Pre-Study Set Up

#### Outcome of Engagement With Community Pharmacies

All 12 pharmacies across the three states that were approached agreed to participate and to undertake training and structural changes to meet clinical trial pharmacy requirements. Study pharmacies across the three states comprised five community pharmacies and three hospital based clinical trials pharmacies in Victoria, one community pharmacy, and one hospital based clinical trials pharmacy in South Australia, and two community pharmacies in Tasmania. At baseline, all hospital based clinical trial pharmacies met GCP requirements. The mean distance from the community pharmacies to the closest PrEPX study clinic was 325 meters (range: 50 m–2 km) (**Table 2** and **Supplementary Figures 6** and **7**).

**Table 2:** Summary of pharmacy study logs used in the pre-exposure prophylaxis expanded study.

	Prescriber training log	Pharmacy training log	Prescriber delegation log	Pharmacy delegation log	Drug accountability log	Participant log
**Completed by**	Clinic staff and study physicians/prescribers	Pharmacists	Study physicians/prescribers	Pharmacists	Pharmacists	Pharmacists
**Completed when**	Clinic site initiation	Pharmacy site initiation	Clinic site initiation	Pharmacy site initiation	Every time drug is delivered and for each dispensing	The first time a participant attends PrEPX dispensing pharmacy
**Site name**	X	X	X	X	X	X
**Date**	X	X	X	X	X	X
**Name**	X	X	X	X		X (Participant and pharmacist)
**Title**	X	X	X	X	–	–
**Signature of**	Prescriber(s)	Pharmacist(s)	Prescriber(s)	Pharmacist(s)	Dispensing pharmacist	Dispensing pharmacist
**Study role**	X	X	X	X	–	–
**PI Authorization**	X	–	X	X	–	–
**Participant ID**	–	–	–	–	X	X
**Participant DOB/MRN**	–	–	–	–	–	X
**Consent checked**	–	–	–	–	–	X
**Study visit no.**	–	–	–	–	X	X
**Drug quantity (bottle)**	–	–	–	–	X	–

#### Structural Improvements

None of the eight community pharmacies had temperature monitors. Eight testo temperature monitors were installed in each pharmacy. During the course of the study, there were 26 temperature alarms, most of which occurred during winter, over the weekend, or when pharmacies were closed. All temperature alarms were promptly rectified within 1 h. The 1-h time frame was insufficient for study drug to equilibrate to the alarmed temperature.

Two of the eight (17%) community pharmacies had lockable storage. Lockable storage was purchased for the remaining six community pharmacies and installed in the restricted access dispensing area, in accordance with GCP study drug storage procedures.

#### Training

None of the eight (0%) community pharmacies had pharmacists with current GCP licenses. All of the hospital based clinical trial pharmacists (100%) had current GCP licenses. As a result, 54 GCP licenses were completed by community pharmacists across eight PrEPX study pharmacies.

### Dispensing

**Figure 2:**
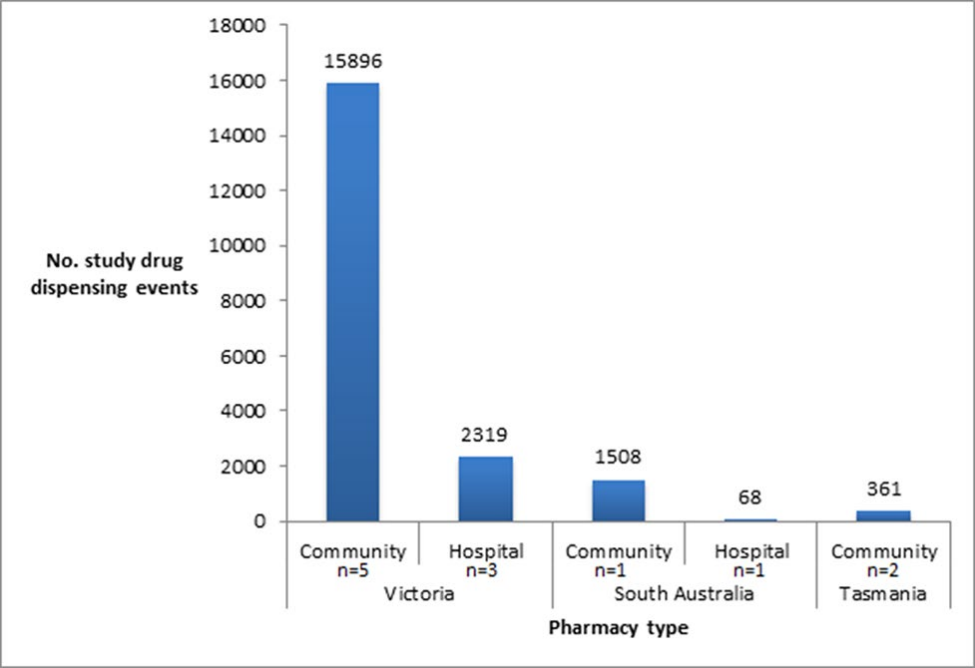
Number of prescriptions dispensed in participating PrEPX Community and Hospital pharmacies.

A total of 60,456 bottles of co-formulated tenofovir disoproxil fumarate and emtricitabine, containing 30 tablets were purchased from Mylan over the course of the PrEPX study. In total, 20,152 prescriptions were dispensed across the twelve PrEPX dispensing pharmacies during the study periods: (Victoria: 18,188; South Australia: 1576; Tasmania: 361). Of those, 16,724 prescriptions (83%) were dispensed by community pharmacies (**Figure 2**). The number of participants seen at each site is not known as participants were free to attend more than one study pharmacy for dispensation.

### Monitoring Outcomes

#### Dispensing Errors Identified Through Monitoring

**Figure 3:**
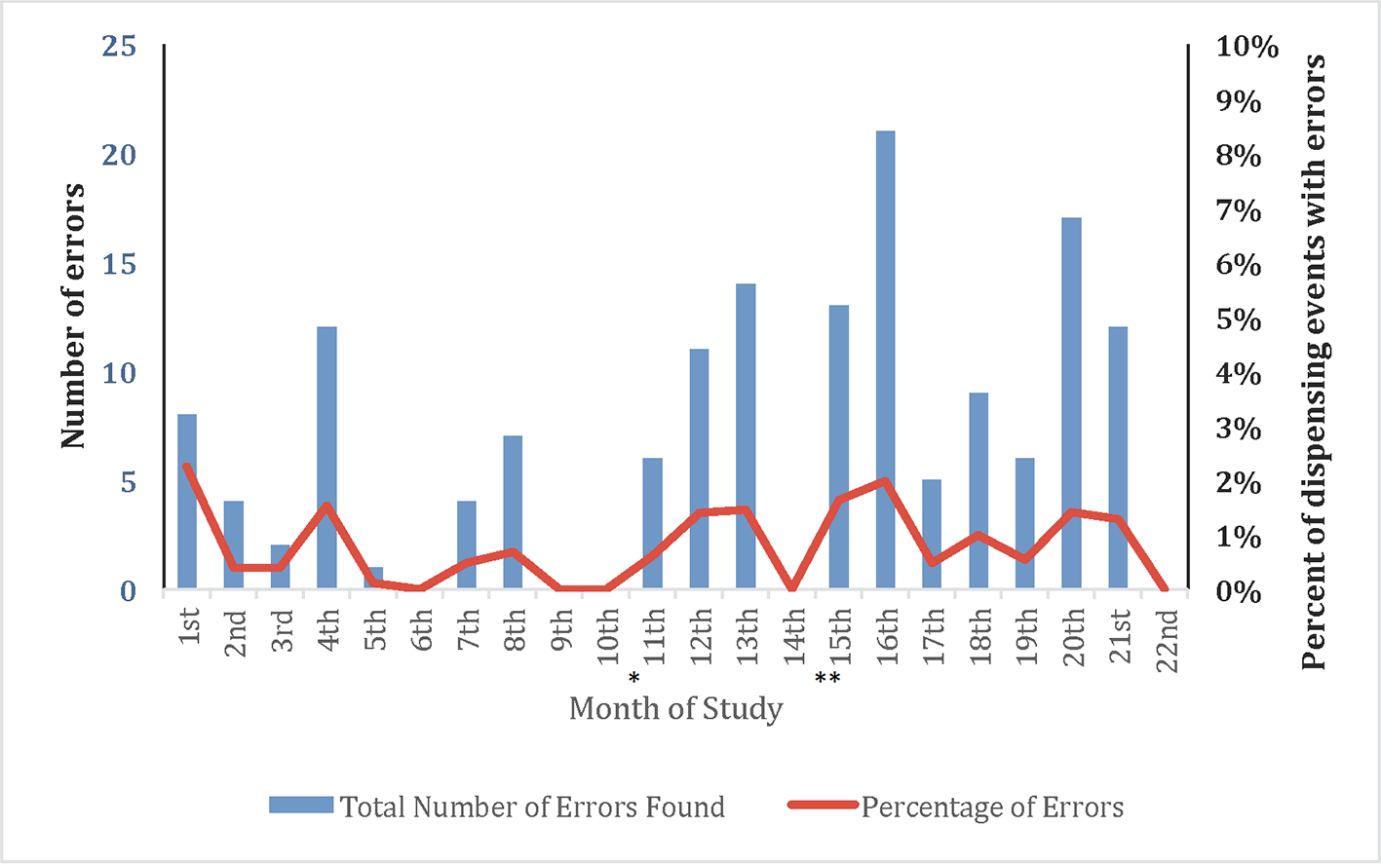
Number of pharmacy errors across all PrPEX study pharmacies in relation to the number of prescriptions dispensed (26 July 2016–30 June 2018). Community 91.5% (n = 375); Hospital 8.5% (n = 35). *South Australian sites open **Tasmanian sites open.

Monitoring visits revealed that the number of action items for follow-up at each participating community pharmacy were greater at the beginning of the study and declined during the first 12 months but rebounded during the following 10 months. The number of action items in a 1-month period ranged from 0 (0%) to 22 (2%), and the greatest number of action items was recorded in study month 18 (**Figure 3**). Action items during the study period included verification of a prescriber code or signature (n = 119) and participant details documented incorrectly, or missing from prescription e.g. DOB and verification of study participant details e.g. study number, confirmation of consent (n = 291). A total of 410 dispensing errors [91.5% (n = 375) community; 8.5% (n = 35) hospital] were identified out of 20,152 (2.0%) of dispensed study prescriptions. Study logs were checked for accuracy and a stock balance check was performed at each monitoring visit. During the course of the study, there were 19 incorrect balance checks observed on the drug accountability logs, which were all attributable to recorded documentation errors. There were no errors noted with the participant logs, or any of the site signature or delegation logs. The number of errors increased when additional study places were released and when new study sites were established (**Figure 3**).

Over the study period the chief items that required follow-up were dispensing signature box not completed (n = 119), prescriptions dispensed outside of the 7/21 day window period (n = 345) and consent confirmation not documented on the prescription (n = 291). Most items that required follow-up occurred at the PrEPX community pharmacies and were able to be rectified in a timely manner (see **Supplementary Figure 2** detailed list of monitoring check items).

#### Dispensing Without Good Clinical Practice Certificate

There were five occasions in which locum or relief pharmacists working in community pharmacies dispensed study drug despite not having the GCP certification. The pharmacy manager provided additional training and discussed in detail any events arising from locum or relief pharmacists with the PrEPX authorized site pharmacist.

## Discussion

In this large clinical PrEP trial involving over 5,000 participants we showed that it is highly feasible to engage community pharmacies to dispense clinical trial study drug. In this study, over 80% of medications were dispensed by community pharmacies with a low dispensing error rate, which is in accordance with similar published studies (McCormack et al., 2016; AVAC, 2018; Grulich et al., 2018). These data suggest that community pharmacies can be utilized in large clinical trials.

Dispensing of trial medication by community pharmacies for PrEPX was integral to the success of the roll-out of the study whereupon community pharmacies dispensed over 80% of all prescriptions in the PrEPX study. The community pharmacies provided convenient access to participants, which may have improved timely collection of the study drug, augmented study retention (Testo Pty Ltd., 2015), and contributed to a smooth transition for clinicians, participants, and community pharmacies when PrEP was listed on the PBS in April 2018. Furthermore community pharmacy dispensing is likely to have contributed to the recruitment into the PrEPX study wherein 1,000 participants were enrolled in 3 weeks and 2,000 participants were enrolled in 10 weeks (Ryan et al., 2017) which is the fastest recruitment known to have occurred into any PrEP trials to date. In an Australian modeling study, the rapid scale-up of PrEP use was shown to be necessary for PrEP to have a population level impact on reducing HIV transmission (Kirby Institute and the Centre for Social Research in Health, 2017). The United Nations General Assembly has endorsed a declaration to end AIDS by 2030 by scaling up HIV prevention, treatment, and care programs (WHO, 2016). Hence jurisdictions planning to meet this goal will need to offer rapid access to PrEP and if they do so in the setting of a clinical trial, this study’s findings, where over 20,000 dispensing events occurred with only 2.0% of errors suggests that they could plausibly adopt this model to dispense *via* community pharmacies.

Significant investment was needed to achieve successful implementation of clinical trial drug dispensing at community pharmacies for the PrEPX study. Study pharmacies required extensive pre-study phase engagement and ongoing monitoring and support throughout the study. We sought to offset the impost on community pharmacies for their participation in PrEPX by paying for pharmacists to undertake GCP training and accreditation, providing study drug at no cost and allowing retention of dispensing fees for study prescriptions.

The successful implementation of clinical trial drug dispensing through community pharmacies may signal the potential for further community pharmacy dispensing in future clinical drug trials. A recent review reported that only 20% of Australian Clinical Trials met their recruitment deadline, and only 50% of Australian clinical sites met their recruitment requirements (Ernst and Young, 2016). Hence all efforts to increase clinical trial participation should be made including enhancing access to clinical trial drug dispensing. This study demonstrates that community pharmacy dispensing is feasible and it should be further evaluated for its potential to enhance clinical trial recruitment and participant retention.

There are a number of limitations in this paper. We did not collect data from community pharmacists about their experiences with participating in the PrEPX study. However, all pharmacies that were approached agreed to participate, no pharmacies withdrew during the study and high accuracy in dispensing was observed across all sites suggesting participation was acceptable. Additionally, we have not reported on participants’ experiences using the study’s community pharmacies. Furthermore, the findings reported here are specific to the implementation of clinical trial dispensing in Australian community pharmacies and may not be transferrable to international settings with different regulatory and health systems. Finally this was not a randomized study and we did not have a second study arm where participants were randomized to only collect study drug from a hospital clinical trials pharmacy. Therefore we could not evaluate whether clinical trial enrolment and participant retention were different between community and hospital trial pharmacies.

### Conclusions

Clinical trial drug dispensing of PrEP at community pharmacies for a large, rapidly enrolling population level study was highly feasible and practicable. These findings suggest that community pharmacies could be used in large future clinical trials including those delivering HIV prevention strategies.

## The PrEPX Study Team

Edwina Wright, Brian Price, Mark Stoové, Simon Ruth, Colin Batrouney, Michael West, Dean Murphy, John de Wit, Luxi Lal, Jennifer Audsley, Christina Chang, Carol El-Hayek, Anne Mak, Alison Duncan, Joe Sasadeusz, Brent Allan, Michael Whelan, Daniel McPhail, David Wilson, Olga Vujovic, Martin Holt, Chris Williams, Steve Wesselingh, James Ward, Danny Gallant, Alison Ward, Jason Asselin, Tim Spelman, John Timothy Lockwood, Alistair Chong, Katharine McKinnon, Kathleen Ryan, Michael Traeger, Christopher Fairley, Ivette Aguirre, Ban Kiem Tee, Norman Roth, Vincent Cornelisse, Timothy Read, Richard Moore, Jeff Willcox, George Forgan-Smith, John Gall, Matthew Penn, Helen Lau, Danielle Collins, Sian Edwards, Susan Boyd, Claire Pickett, Emma Paige, Pauline Cundill, Amanda Wade, Charlotte Bell, William Donohue, Samuel Elliot, Helen Calabretto, Louise Owen.

## Data Availability Statement

The raw data supporting the conclusions of this manuscript will be made available by the authors, without undue reservation, to any qualified researcher.

## Ethics Statement

This project was approved by The Alfred Hospital Health Research Ethics Committee for the study to be conducted in Victoria and South Australia (AH/HREC16/100). Separate ethics approval was sought and granted from The University of Tasmania for The PrEPX Study to be conducted in Tasmania (H0016607). The study was registered on the Australian and New Zealand Clinical Trials Registry (ACTRN12616001215415).

## Author Contributions

EW, AM and LL contributed conception and design of the study; IL, TL and LL organized the database; LL and KR performed the statistical analysis; LL, KR, AM and EW wrote the manuscript. All authors contributed to manuscript revision, read and approved the submitted version.

## Funding

This study was funded by the Victorian Department of Health, Victoria, Australia; Alfred Health, Melbourne, Australia; The Victorian AIDS Council, Melbourne, Australia; The Government of South Australia, Australia; The Department of Health and Human Services, Tasmania, Australia.

## Conflict of Interest

EW has received financial support from Gilead Sciences; Abbott Laboratories; Janssen-Cilag; Boehringer Ingelheim; ViiV Healthcare; and Merck Sharp & Dohme. Gilead Sciences donated study drug to the VicPrEP study (precursor to the PrEPX study). Authors MV and KL were employed by the company Prahran Central Pharmacy, South Yarra, Victoria, Australia. Author JS was employed by the company John Silverii’s Pharmacy, Fitzroy North, Victoria, Australia. Author JT was employed by the company HealthSmart Pharmacy at The Alfred, Melbourne, Victoria, Australia. Authors JP and WL were employed by the company Newton and Leung Pharmacy, Collingwood, Victoria, Australia. Author BN was employed by the company Bain and Co. Pharmacy, St Kilda, Victoria, Australia. Author NR was employed by the company Centre Pharmacy, Adelaide, South Australia. Authors MR, RS and CC were employed by the company Epic Pharmacy, New Town, Hobart, Tasmania. Authors AH and MQ were employed by the company Epic Pharmacy Kings Meadows, Launceston, Tasmania. 

The remaining authors declare that the research was conducted in the absence of any commercial or financial relationships that could be construed as a potential conflict of interest.
