# Supplementary material for: Transformation of Australian Community Pharmacies Into Good Clinical Practice Compliant Trial Pharmacies for HIV Pre-Exposure Prophylaxis
Source: Front Pharmacol. 2019 Nov 7;10:1269. doi: 10.3389/fphar.2019.01269 (PMC6854879; doi:10.3389/fphar.2019.01269)
Supplement: Supplementary file 1 [file DataSheet_1.pdf]

COMMUNITY PHARMACY CLINICAL TRIALS PRESCRIPTION

**Subject's Full Name:**<br>*(Handwritten by Study Doctor)*	**DOB:** / /<br>DD / MM / YYYY
**Address:**	
**Allergies:**	**Consent Obtained:**<br>YES / NO
**Medicare Number:**	**Exp:**
**Concession Card No.**<br>**(if applicable)**	**Exp:**
**Protocol: PrEPX**	**Alfred Ethics Project: AH 100/16**
**Participant ID:**	**Prescriber:**<br>*(First and Last Names)*
**Visit No: Month ___**	**Treatment Date:**

Tenofovir Disoproxil Fumarate 300mg / Emtricitabine 200mg oral tablets

Take ONE tablet orally ONCE DAILY, with or without food.

Dispense 3 bottles (30 tablets per bottle)

Prescriber's Signature: ______________________________ Prescribing Date: ___________

Dispensed by (P = pharmacist only)		
	Signature	Date
Dispensing record checked by (P)		
Prescriber's name and signature in delegation log checked by		
Dispensed medications Checked by (P)		
Handed out by (P)		
Received by: Patient or Patient Delegate		

Attach pharmacy dispensing label

Version 3.0 dated 08FEB2017 Prepared by: LL Checked by: MAK

Supplementary Figure A: PrEPX PRESCRIPTION PROFORMA

PrEPX Pharmacy Monitoring Checklist

Name of Pharmacy: ___________________________________

Monitor Name: __________________________

**Description**	**No. of errors**	**Corrected/ Action/Who**
Consent checked		
Study ID recorded		
Prescription signed by authorised PrEPX prescriber		
Date collected by participant within 21 days of written/dated prescription (3,6,9 month etc.)		
Date collected within 7 days for NEW participants (baseline only)		
Dispensing check box initialled and dated correctly		
Initials of GCP certified pharmacist		
All entries initialled and dated correctly in accountability log		
Stock count		
Batch numbers and page numbers filled out on accountability log		
Single entries on accountability log		
Logs in date order		
Prescriptions filed in numerical order in correct folder		
Stock kept in locked cabinet		
Temperature records checked		

Monitor Signature___________________________ **Date** ___ / ___ / ____

Supplementary Figure B: PrEPX Pharmacy Monitoring Checklist

INVESTIGATIONAL PRODUCT NAME	STUDY PROTOCOL	INVESTIGATOR NAME	INSTITUTION / SITE NAME
Tenofovir Disoproxil Fumarate 300mg / Emtircitabine 200mg	The PrEPX Study Alfred Ethics Project 100/16	A/Prof Edwina J Wright	Happy Valley Pharmacy

Site Delegation and Signature Log

Print Full Name & Title	Signature	Initials	*Study Role	**Key Delegated Study Task(s) See list below	Duration		Investigator's Authorisation
					From (start-up)	To	
John A Smith	JSmith	J.S.	Pharmacist	13,14	25-07-16	Study end	EJW
Susan Edwards	SCEdwards	S.E.	Pharmacist	13,14	25-07-16	Study end	EJW

*Identification of study role includes but is not limited to sub-investigators, study nurses, pharmacist (when appropriate) and data recorders. List individuals delegated significant study-related tasks (ICH GCP 4.1.5). Signature/Initials required for all persons authorised to make entries and/or corrections to Case Report Forms (ICH GCP 8.3.24) ** Identify key study tasks when delegated by the investigator. Examples of key study tasks include:

1	Informed Consent collection	7	Handling of blood samples	13	Investigational Product dispensing	19	Other (specify):
2	Medical History review	8	Review of incl./exclusion criteria	14	Investigational Product Handling	20	Other (specify):
3	Concomitant Meds review	9	Safety assessments	15	CRF Completion	21	Other (specify):
4	Physical Exams administration	10	Efficacy assessments	16	CRF Signature	22	Other (specify):
5	Measure of vital signs	11	Authorisation to randomise	17	Data Query Completion	23	Other (specify):
6	Collection of blood samples	12	IVRS/IWRS procedures	18	Data Query Signature	24	Other (specify):

Supplementary Figure C: PrEPX Signature and Delegation Log

STUDY PROTOCOL	INVESTIGATOR NAME	INSTITUTION / SITE NAME
**The PrEPX Study**<br>**Alfred Ethics Project 100/16**		

Page_____of ______

Participant Master Log

Date	Participant Name	Participant DOB/MRN	Participant No.	Consent Obtained YES/NO	Signature	Comments

Supplementary Figure D: PrEPX Pharmacy Participant Log

INVESTIGATIONAL PRODUCT NAME	STUDY PROTOCOL	INVESTIGATOR NAME	INSTITUTION / SITE NAME
**Tenofovir Disoproxil Fumarate 300mg / Emtricitabine 200mg**	**The PrEPX Study Alfred Ethics Project 100/16**	**A/Prof Edwina J Wright**	**Happy Valley Pharmacy**

Drug Accountability Log

DRUG: Tenofovir Disoproxil Fumarate 300mg / Emtricitabine 300mg oral tablets	PACKSIZE: 30 tablets per bottle
Batch: *403486 PPP106/2017* (One Batch per page)	Expiration Date: *June 2020*

Receipt			Dispensing					Balance (bottle)	Returns			Checked/ signed by PrEPX pharmacy manager
Date	Quantity (bottle)	By	Date	Visit	Participant ID	Quantity (bottle)	Dispensed by		Date	Quantity (bottle)	Received By	
04-06-17	*300*	*JS*										*JSmith*
			04-06-17	*0*	*XYZ1234*	*3*	*JS*	*297*				*JSmith*

Supplementary Figure E: PrEPX Pharmacy Accountability Log

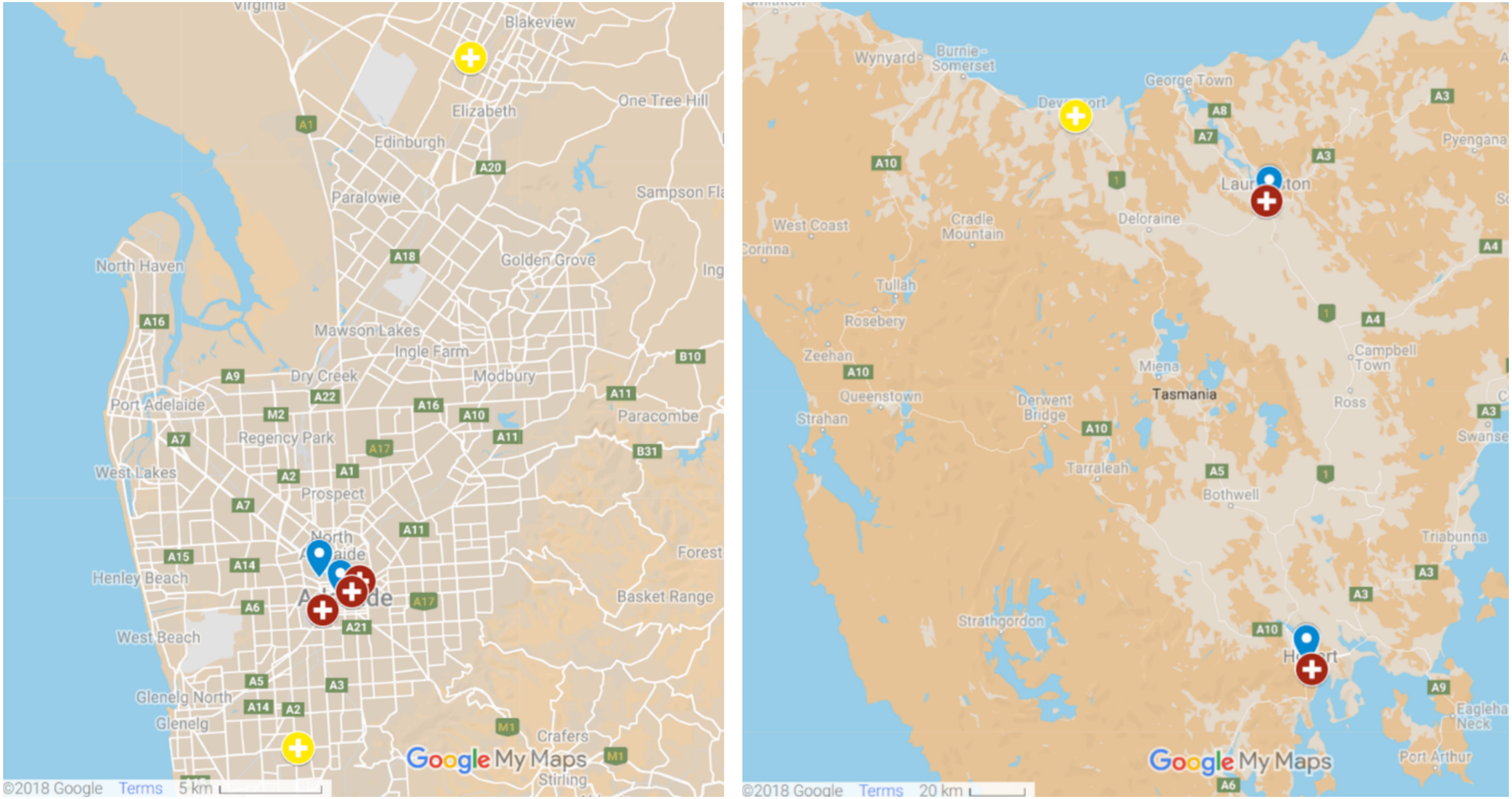

Supplementary Figure F: Geographical locations of South Australian (L) and Tasmanian (R) participating PrEP clinics, PrEP dispensing pharmacies and clinics where PrEP medication was posted to participants due to limited pharmacy accessibility.

Key:

- PrEP prescribing clinic- red circle cross
- Dispensing pharmacy- blue droplet
- PrEP postal service clinic- yellow circle cross

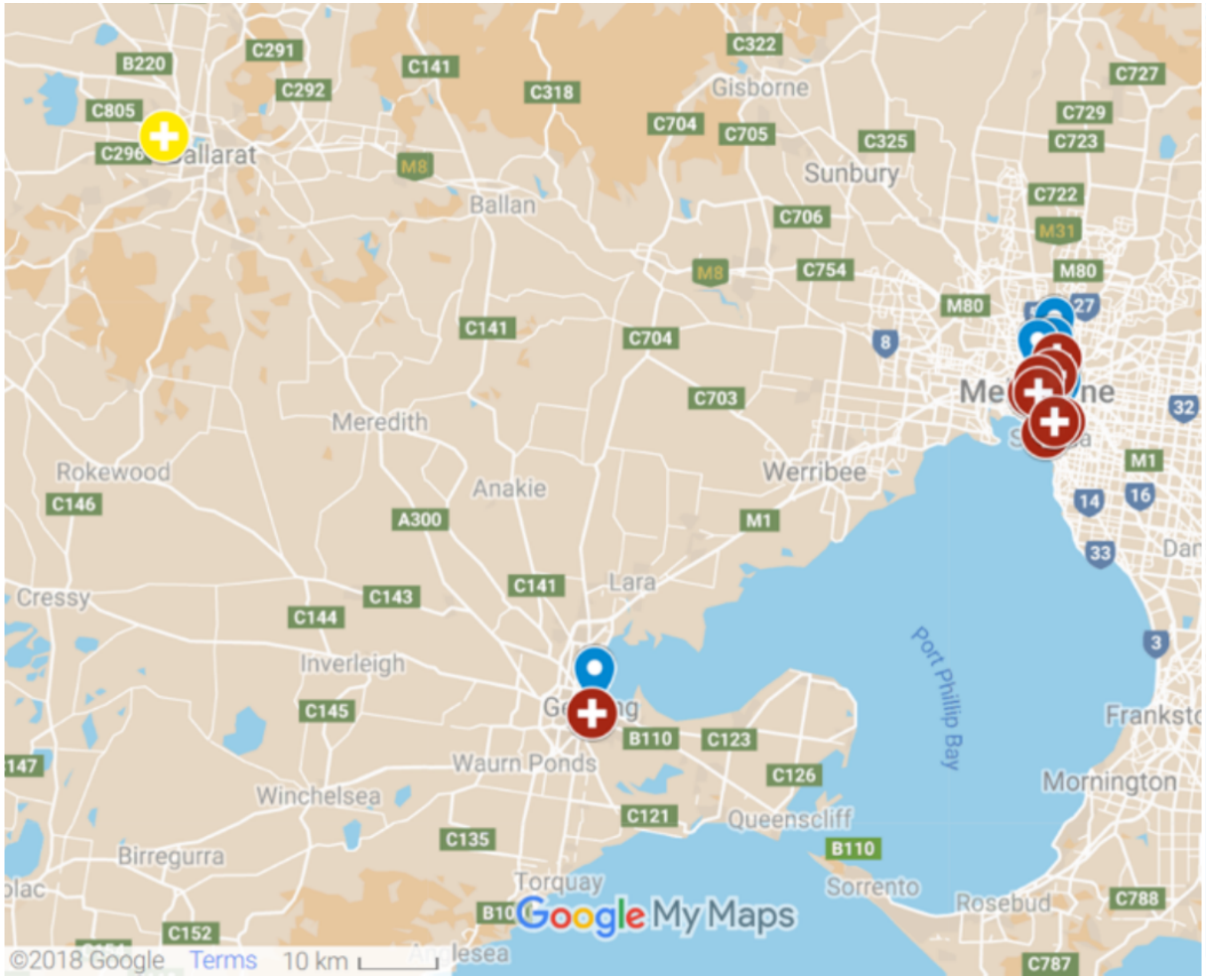

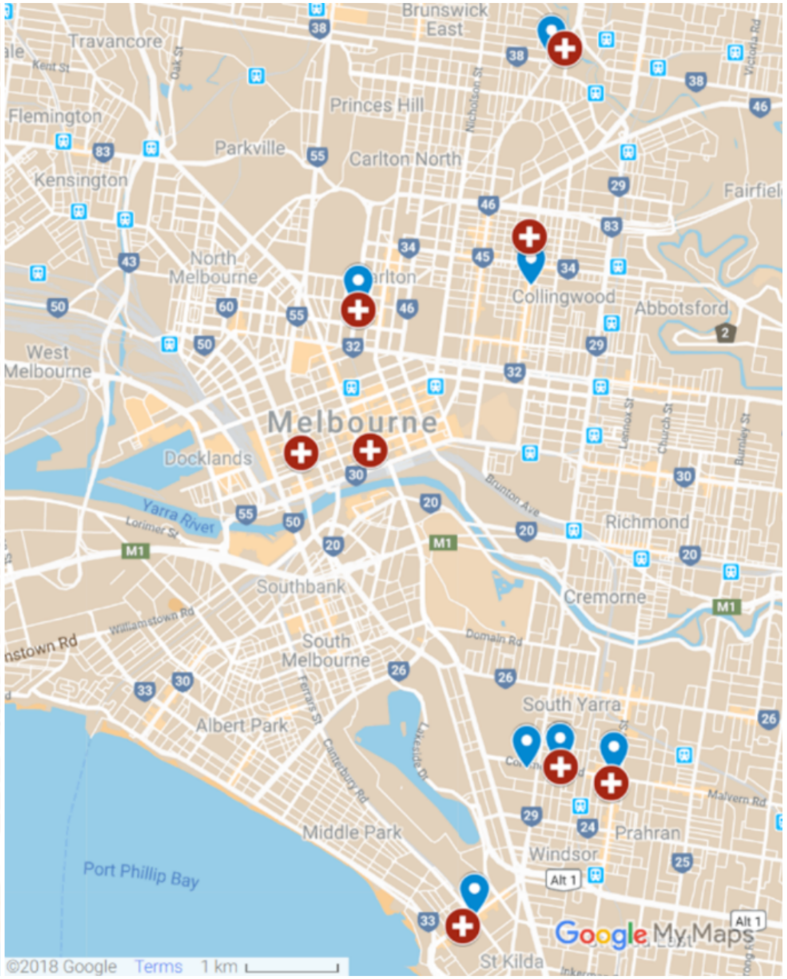

Supplementary Figure G: Geographical locations of Victorian participating PrEP clinics, PrEP dispensing pharmacies and clinics where PrEP medication was posted to participants due to limited pharmacy accessibility.

Key:

- **PrEP prescribing clinic- red circle cross**
- **Dispensing pharmacy- blue droplet**
- **PrEP postal service clinic- yellow circle cross**
